# Results of Dynamic Contrast-Enhanced Ultrasound Correlate With Treatment Outcome in Canine Neoplasia Treated With Electrochemotherapy and Interleukin-12 Plasmid Electrotransfer

**DOI:** 10.3389/fvets.2021.679073

**Published:** 2021-05-20

**Authors:** Maja Brloznik, Simona Kranjc Brezar, Nina Boc, Tanja Knific, Maja Cemazar, Nina Milevoj, Gregor Sersa, Natasa Tozon, Darja Pavlin

**Affiliations:** ^1^Veterinary Faculty, Small Animal Clinic, University of Ljubljana, Ljubljana, Slovenia; ^2^Department of Experimental Oncology, Institute of Oncology Ljubljana, Ljubljana, Slovenia; ^3^Faculty of Medicine, University of Ljubljana, Ljubljana, Slovenia; ^4^Department of Radiology, Institute of Oncology Ljubljana, Ljubljana, Slovenia; ^5^Institute of Food Safety, Feed and Environment, Veterinary Faculty, University of Ljubljana, Ljubljana, Slovenia; ^6^Faculty of Health Sciences, University of Primorska, Izola, Slovenia; ^7^Faculty of Health Sciences, University of Ljubljana, Ljubljana, Slovenia

**Keywords:** dog, contrast-enhanced ultrasonography, electrochemotherapy, immunotherapy, gene electrotransfer, bleomycin, cisplatin, IL-12 plasmid

## Abstract

Electrochemotherapy (ECT) and/or gene electrotransfer of plasmid DNA encoding interleukin-12 (GET pIL-12) are effective treatments for canine cutaneous, subcutaneous, and maxillofacial tumors. Despite the clinical efficacy of the combined treatments of ECT and GET, data on parameters that might predict the outcome of the treatments are still lacking. This study aimed to investigate whether dynamic contrast-enhanced ultrasound (DCE-US) results of subcutaneous tumors differ between tumors with complete response (CR) and tumors without complete response (non-CR) in dogs treated with ECT and GET pIL-12. Eight dogs with a total of 12 tumor nodules treated with ECT and GET pIL-12 were included. DCE-US examinations were performed in all animals before and immediately after therapy as well as 8 h and 1, 3, and 7 days later. Clinical follow-up examinations were performed 7 and 14 days, 1 and 6 months, and 1 year after treatment. Numerous significant differences in DCE-US parameters were noted between tumors with CR and non-CR tumors; perfusion and perfusion heterogeneity were lower in CR tumors than in non-CR tumors. Therefore, studies with larger numbers of patients are needed to investigate whether DCE-US results can be used to predict treatment outcomes and to make effective decisions about the need for repeated therapy or different treatment combinations in individual patients.

## Introduction

Dynamic contrast-enhanced ultrasound (DCE-US) is a simple, readily available, non-invasive, safe, and inexpensive method for assessing tissue perfusion at the capillary level and correlates with histological results of vessel density in preclinical (1–3) and clinical studies (4–7). Moreover, DCE-US has been used to predict the efficacy of antiangiogenic treatments of various tumors in preclinical (3) and human clinical trials (8–17), whereas DCE-US had no predictive value in canine tumors treated with radiotherapy (18). Furthermore, DCE-US results correlated with the results of advanced diagnostic imaging (19). The contrast agents used in DCE-US are gas-filled microbubbles stabilized in a lipoprotein shell that have a diameter of 1–3 μm, which is small enough to migrate freely through the circulation and large enough to remain in the vascular space (20–22). Capillary filling results in diffuse enhancement of perfused tissue. Most of the contrast agent is excreted through the lungs within 20 min after administration (23). The advantages of contrast agents used in DCE-US over those used in dynamic contrast-enhanced computed tomography (DCE-CT) are numerous: the contrast agents allow real-time imaging, there is no ionizing radiation, they are neither nephro- nor hepatotoxic, and they have very few, very mild side effects (22–24). In humans, contraindications to microbubble administration include pulmonary hypertension and impaired cardiopulmonary function (24). In a large number of dogs in which DCE-US examinations were performed, <1% developed an immediate effect, including vomiting and/or syncope, or delayed adverse effects, including vomiting (23).

Electrochemotherapy (ECT) and/or gene electrotransfer of plasmid DNA encoding interleukin-12 (GET pIL-12) are effective treatments for cutaneous, subcutaneous and maxillofacial tumors in dogs (25–41), superficial cell carcinoma in cats (42), cutaneous tumors in ferrets (43) and sarcoid tumors in horses (44, 45). Several preclinical (46–49) and clinical studies in veterinary patients (26–28, 33, 36, 50) have shown that the effect of ECT is potentiated by GET pIL-12, and ECT has become an established standard of care for a variety of human cancers: cutaneous and subcutaneous tumors, including melanoma, squamous cell carcinoma, basal cell carcinoma, and other metastases (51–58); hepatocellular carcinoma and colorectal liver metastases (59–63); pancreatic neoplasia (64–66); and others. A portion of the antitumor efficacy of electroporation (EP)-based therapies arises from the effect of EP on the vasculature of the treated tumor, inducing a local blood flow effect, namely, “vascular lock,” i.e., small blood vessel vasoconstriction and increased wall permeabilization (67–74). The vascular effects of EP are enhanced by the use of chemotherapeutic agents in ECT treatment, and the effect lasts longer in tumors than in healthy tissue, namely, the “vascular disrupting effect” (67, 68, 72).

Despite the clinical efficacy of the combined treatments of ECT and GET pIL-12, there is still a lack of data on parameters that might predict the outcome of the treatments. For EP-based treatments, it could be assumed that the therapy should be repeated if it does not reflect in the expected “vascular lock” immediately after the treatment and/or anti-angiogenic effects in the days after.

This pilot study aimed to investigate whether the DCE-US results from subcutaneous tumors correlate with treatment outcomes in dogs treated with ECT combined with GET pIL-12.

## Materials and Methods

### Design and Setting

Eight dogs (seven females and one male) with a total of 12 superficial tumor nodules (11 mast cell tumors and 1 neurofibrosarcoma) treated with ECT and GET pIL-12 were included (Table 1). Their mean age with standard deviation was 8.0 ± 2.3 years. Six dogs had one tumor, and two dogs had three tumors. Each nodule was measured in three perpendicular directions (a, b, c), and tumor volume was calculated using the formula: V = a × b × c × π/6. Owners of the dogs signed an informed consent form before inclusion.

**Table 1:** Characteristics of the eight dogs treated with electrochemotherapy (ECT) combined with gene electrotransfer of plasmid DNA encoding canine interleukin-12 (GET pIL-12).

**Patient no**.	**Age**	**Sex**	**Breed**	**Weight (kg)**	**Tumor**	**No of tumors**	**Tumor volume (cm^**3**^)**
1	9 y 11 m	Female	Mixed breed	25.0	Mast cell tumor	1	0.38
2	9 y 7 m	Female	German hunting terrier	11.3	Mast cell tumor	3	0.04; 0.004; 0.01
3	9 y 10 m	Female	Beagle	17.3	Neurofibrosarcoma	1	23.00
4	10 y 8 m	Female	Mixed breed	34.4	Mast cell tumor	3	1.10; 0.10; 0.004
5	6 y 5 m	Female	Basset hound	29.0	Mast cell tumor	1	5.00
6	4 y 10 m	Female	Golden retriever	34.2	Mast cell tumor	1	0.92
7	5 y 11 m	Male	Boston terrier	10.7	Mast cell tumor	1	0.37
8	6 y 7 m	Female	Bernese mountain dog	40.3	Mast cell tumor	1	0.27

### ECT Combined With GET pIL-12

The procedures were performed under general anesthesia: the dogs received 0.2 mg/kg midazolam (Midazolam Torrex, Torrex Pharma GesmbH, Vienna, Austria) intravenously, and anesthesia was induced by 3–6 mg/kg propofol (Diprivan, Zeneca, Grangemouth, United Kingdom) administered intravenously and maintained with isoflurane (Isoflurin, Vetpharma Animal Health, Barcelona, Spain). All patients received fluid therapy throughout the procedure by administering Hartmann's solution (B Braun Melsungen AG, Melsungen, Germany) at a rate of 5 mL/kg/h.

An electrical pulse generator, CliniporatorTM (IGEA s.r.l., Carpi, Italy), was used to deliver electrical pulses through either plate, hexagonal, or needle electrodes. The selection of electrode type, voltage, duration, and frequency of the electrical pulses was based on ECT (23, 35, 48–52) and GET studies (25–28, 33) (Table 2).

**Table 2:** Electrochemotherapy (ECT) combined with gene electrotransfer of plasmid DNA encoding canine interleukin-12 (GET pIL-12) treatment regimens (dosages, route of administration, type of electrodes and pulse parameters) and outcome per patient.

**Patient no**.	**ECT**					**GET pIL12**			**Outcome**
	**Drug**	**Dose (mg)**	**Administration**	**Type of electrodes**	**Pulse parameters**	**pIL-12 administration (2 mg)**	**Type of electrodes**	**Pulse parameters**	
1	BLM	6.90	i.v.	Plate	ECT pulses	p.t.	MEA	GET pulses	CR
2	CDDP	T1: 0.60	i.t.	Plate	ECT pulses	p.t.	MEA	GET pulses	Non-CR (PD)
		T2: 0.20							Non-CR (PD)
		T3: 0.20							CR
3	CDDP	12.00	i.t.	/		i.t.	Hexagonal	ECT pulses	Non-CR (PR)
4	BLM	10.47	i.v.	/		i.t.	Hexagonal	ECT pulses	Non-CR (PD)
									CR
									CR
5	CDDP	5.00	i.t.	/		i.t.	Hexagonal	ECT pulses	CR
6	CDDP	0.90	i.t.	/		i.t.	Hexagonal	ECT pulses	CR
7	CDDP	0.37	i.t.	/		i.t.	Needle	ECT pulses	Non-CR (PR)
8	CDDP	0.27	i.t.	/		i.t.	Plate	ECT pulses	CR

*BLM, bleomycin; CDDP, cisplatin; i.v., intravenously; i.t., intratumorally; p.t., peritumorally; T, tumor nodule; ECT pulses, 8 pulses of 100 μs duration with an amplitude to electrode distance ratio of 1,300 V/cm and a frequency of repetition of 5 kHz; GET pulses = 150 ms pulse of amplitude 170 V/cm; / = electroporation was performed simultaneously for ECT and GET; CR, tumor with complete response; MEA, multielectrode array; non-CR, tumor without complete response; PD, progressive disease; PR, partial response*.

For the ECT procedure, two dogs received bleomycin (Blenoxane, Bristol-Myers, NY, USA) at a concentration of 3 mg/mL intravenously at a dose of 0.3 mg/kg, and six dogs received cisplatin (cis-diammine dichloroplatin II, Cisplatin Accord 1 mg/mL, Accord Health Care, Warsaw, Poland) at a concentration of 1 mg/mL and at a dose of 1 mg/cm^3^ intratumorally (Table 2).

For the GET procedure, the pCMVcaIL-12 plasmid encoding canine IL-12 was used, isolated using the Qiagen Endo-Free kit (Qiagen, Hilden, Germany), and diluted to a concentration of 1 mg/mL in endotoxin-free water (Qiagen). Quality control and quantification were performed (28). The plasmid was injected at a dose of 2 mg per patient (27, 28, 33) peritumorally in two dogs and intratumorally in six dogs (Table 2). When more than one tumor was present in a patient, the dose of pIL-12 was divided among the tumors proportional to the tumor volume (Table 2).

### DCE-US

**Figure 1:**
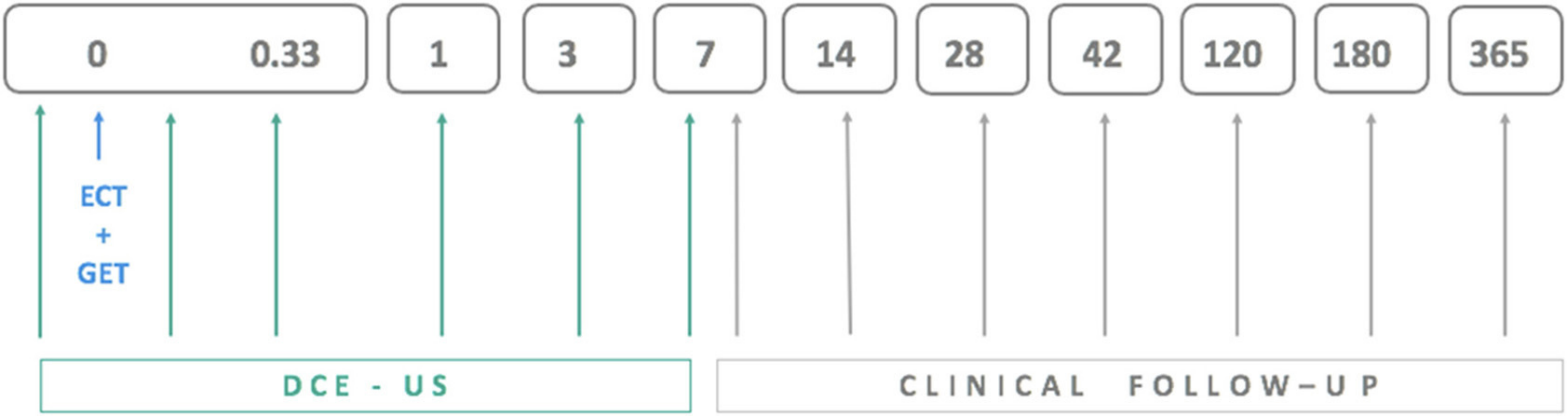
Schedule for electrochemotherapy combined with gene electrotransfer of plasmid DNA encoding interleukin IL-12 (ECT+GET), dynamic contrast-enhanced ultrasound (DCE-US) examinations and clinical follow-up examinations, which included tumor volume measurement.

DCE-US examinations were performed in all animals before and immediately after therapy as well as 8 h and 1, 3, and 7 days later (Figure 1). For the first two measurements, the dogs were under general anesthesia (described above for ECT combined with GET) but were awake for the DCE-US measurements at 8 h and 1, 3, and 7 days after therapy. The contrast agent Sonovue (Bracco, Milan, Italy) was administered into the cephalic vein at dose 0.5–1.5 mL per dog, depending on the weight of the animal (<10 kg: 0.06 mL/kg, 10–20 kg: 0.05 mL/kg, 20–30 kg: 0.04 mL/kg, and >30 kg: 0.03 mL/kg).

Ultrasound examinations were performed with a Resona 7 ultrasound scanner and a linear probe L11-3u with a frequency of 3–10 MHz (Mindray, Shenzhen, China). Low mechanical index harmonic non-linear ultraband contrast imaging was used. From the time of contrast application, a 90-s recording was made. The Dicom files of the examinations were imported into the free software Vuebox^TM^ (Bracco, Milan, Italy) to quantify tissue perfusion with DCE-US. Each tumor was carefully delineated, and two additional regions of interest (ROIs), each representing half of the tumor and the reference region representing the tissue below the tumor, were drawn. The arrival time of the contrast agent was manually selected. For each of the perfusion or time-intensity curves representing signal intensity over time, the following was recorded: basic intensity (BI) when no contrast agent was present, peak intensity (PI), and time to peak (TTP) in ms, the time at which the contrast intensity reached its peak. PE (peak enhancement) was calculated as the difference between PI and BI.

The dogs were closely monitored for adverse effects of contrast administration: vomiting, respiratory distress, syncope, nausea, and other effects. The dogs were hospitalized for the first three measurements. For the last three measurements, the dogs were monitored as outpatients for immediate effects (<1 h) and by the owner for delayed effects.

### Clinical Follow-Up Examinations

Clinical follow-up examinations that included measurement of the three perpendicular tumor dimensions were performed at 7 and 14 days, 1 and 6 months and 1 year after treatment (Figure 1). Tumors were classified as having a complete response (CR) or not having a complete response (non-CR) with the latter including partial response (at least 30% decrease in tumor size), progressive disease (>20% increase in tumor size), and stable disease (neither sufficient shrinkage to qualify for partial response nor sufficient increase to qualify for progressive disease) according to RECIST (75) and iRECIST (76) criteria.

### Statistical Analysis

Statistical software R, version 3.6.2, was used for the statistical analysis (77). The parameters of interest are defined in Table 3.

**Table 3:** Definition of tumor volume and dynamic contrast-enhanced ultrasound parameters (DCE-US) and their calculation.

**Parameter**	**Symbol**	**Calculation**
Volume of tumor before the treatment (cm^3^)	V	a × b × c × π/6 (a,b,c = perpendicular tumor dimensions)
Peak enhancement	PE	= Peak Intensity (PI) – Basic Intensity (BI)
Time to peak	TTP	
Ratio between PE of the tumor and PE of the reference (PE_ref_)	*PE ratio*	$=\frac{PE}{\;PE_{ref}}$
Ratio between TTP of the tumor and TTP of the reference	*TTP ratio*	$=\;\frac{TTP}{TTP\;ref}$
Percentage change in PE	*PE ch*	$=\;\frac{PE_{t}}{PE_{t0}}\times 100-100$
Percentage change in TTP	*TTP ch*	$=\;\frac{TTP_{t}}{TTP_{t0}}\times 100-100$
Percentage difference in PE between ROI1 and ROI2	*PE*_*ROI*_ *dif*	$\frac{PE_{ROI1}-PE_{ROI2}}{\left(PE_{ROI1}+PE_{ROI2}\right)/2}\times 100$
Percentage difference in TTP between ROI1 and ROI2	*TTP*_*ROI*_ *dif*	$\frac{TTP_{ROI1}-TTP_{ROI2}}{\left(TTP_{ROI1}-TTP_{ROI2}\right)/2}\times 100$
Percentage difference in change of PE between ROI1 and ROI2	*PE*_*ROI*_ *dif*_*ch*_	$\frac{PE\;ch_{ROI1}-PE\;ch_{ROI2}}{\left(PE\;ch_{ROI1}+PE\;ch_{ROI2}\right)/2}\times 100$
Percentage difference in change of TTP between ROI1 and ROI2	*TTP*_*ROI*_ *dif*_*ch*_	$=\;\frac{TT{P\;ch}_{ROI1}-{TTP\;ch}_{ROI2}}{\left(TT{P\;ch}_{ROI1}-{TTP\;ch}_{ROI2}\right)/2}\times 100$

The normality of the data was tested using the Shapiro Wilk test. Data were not normally distributed; therefore, the comparison between CR and non-CR tumors for each variable was calculated using the Wilcoxon rank-sum test. Statistical significance was set at 5%.

## Results

### Response to Therapy

The size of the tumors varied from 0.004 to 23.0 cm^3^ (median 0.32 cm^3^). The median baseline tumor volume did not differ between CR and non-CR tumors. Furthermore, none of the DCE-US parameters correlated with pretherapy tumor volume.

**Figure 2:**
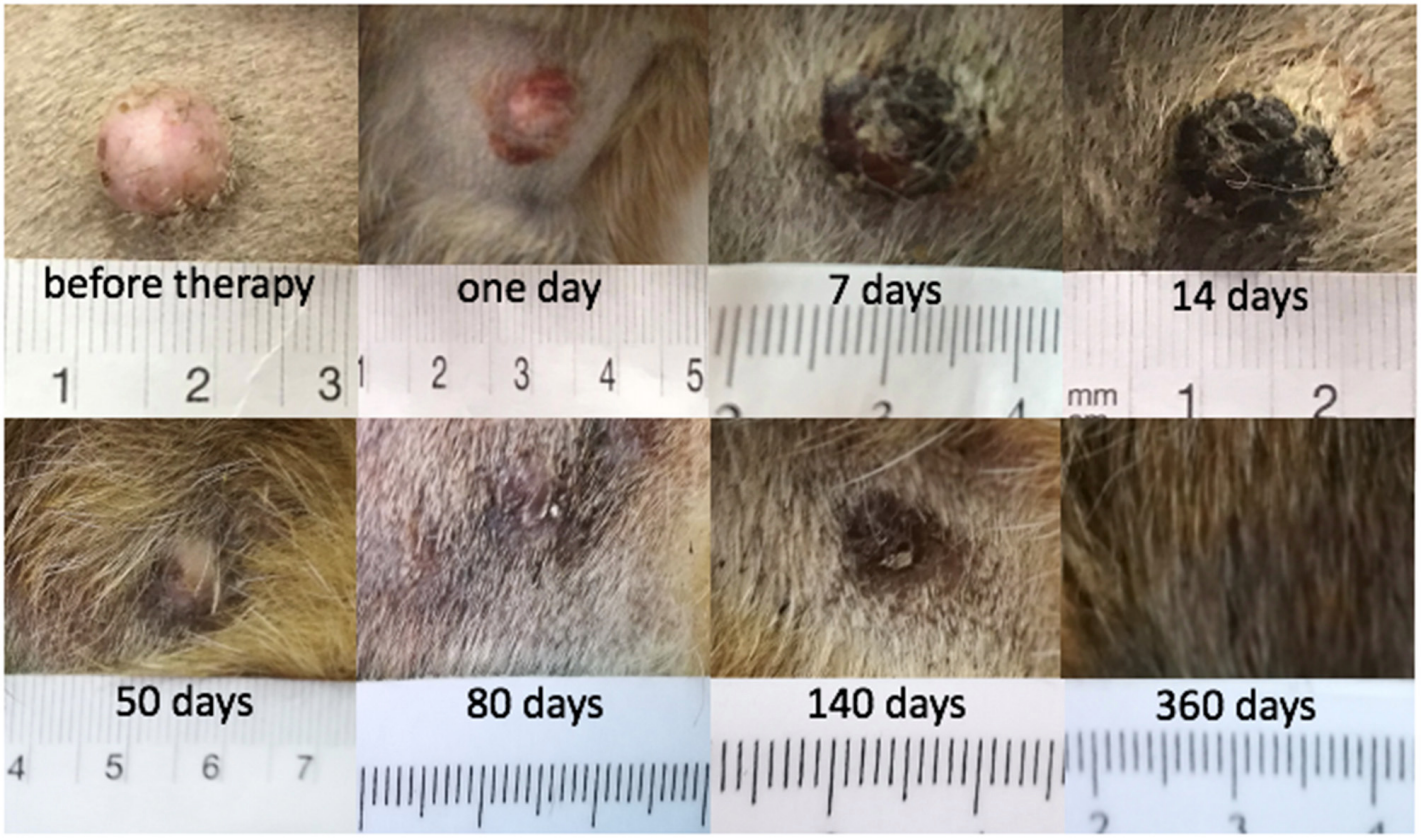
Mast cell tumor in dog 1 where a complete response (CR) was achieved after electrochemotherapy (ECT) combined with gene electrotransfer of plasmid DNA encoding interleukin IL-12 (GET pIL-12).

There were seven tumors with CR in six dogs: dogs 1, 2, 4, 5, 6, and 8. Clinical follow-up examination of the tumor of dog 1 is presented in Figure 2. There were five tumors without CR in four dogs (dogs 2, 3, 4, and 7; Table 2). Three of the non-CR tumors were classified as progressive disease, and two showed a partial response (Table 2).

No adverse effects of contrast administration were noted in our study.

### DCE-US Results

**Figure 3:**
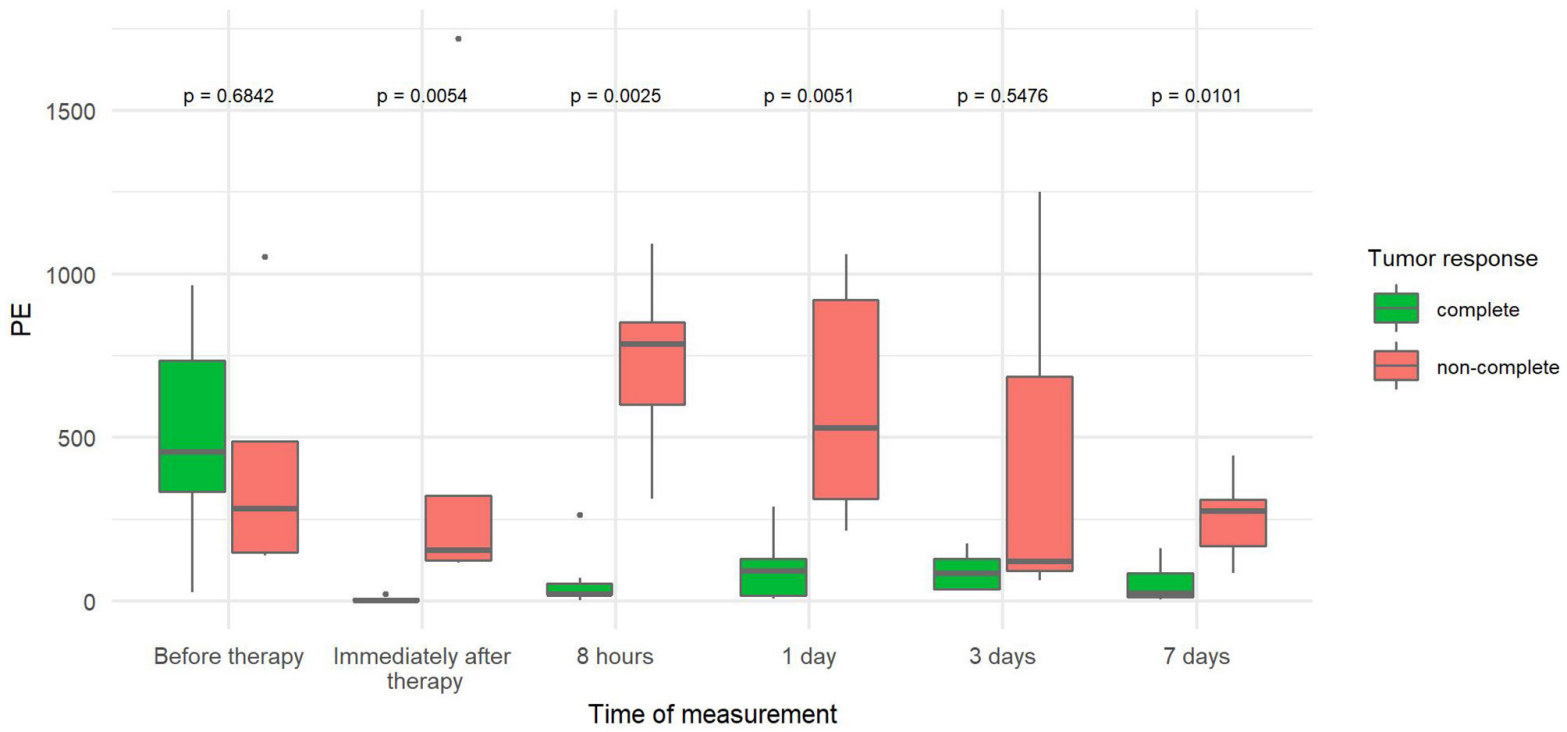
Comparison of peak enhancement (PE) at different time points between canine tumors with complete (CR) and non-complete (non-CR) responses to combined electrochemotherapy treatment with gene electrotransfer of plasmid DNA encoding interleukin IL-12 (ECT GET pIL-12). Note the *p*-values presented at the top of the image. Note that PE is significantly lower for complete response tumors than for non-complete response tumors at all time points after therapy except at 3 days.

**Figure 4:**
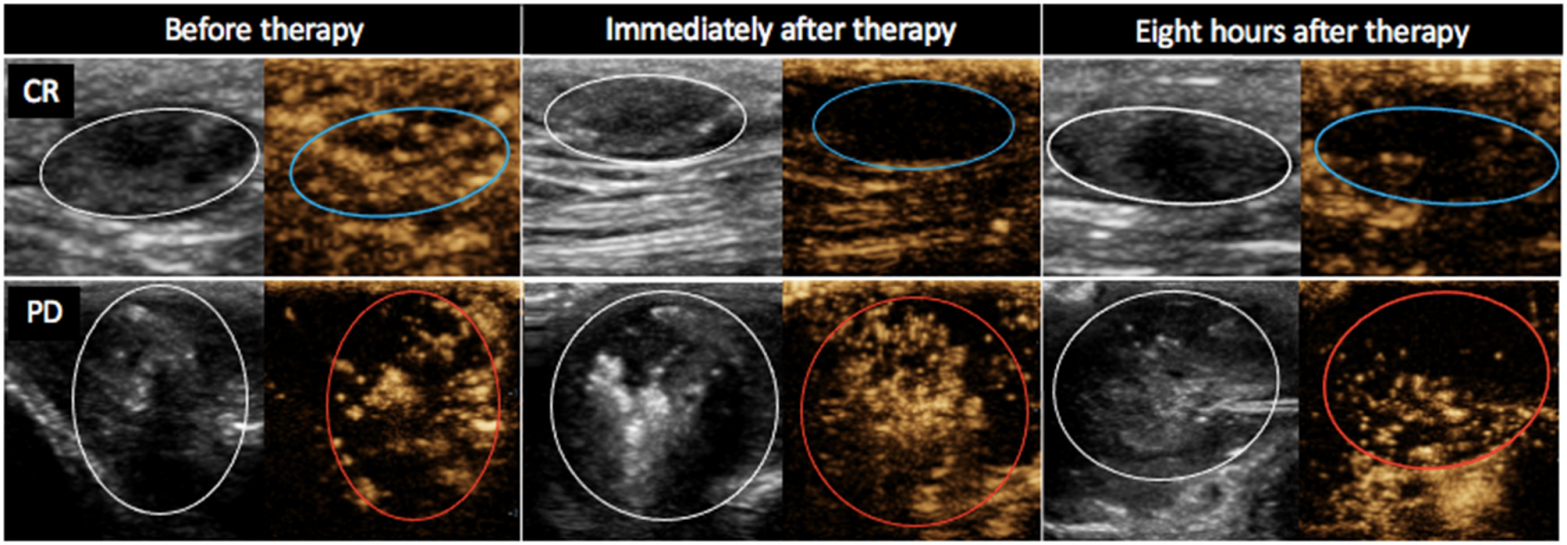
Contrast-enhanced ultrasound images (B mode image to the left and non-linear contrast mode to the right of each image) of two mast cell tumors in dog 4. Images were obtained 20 s after contrast administration at different time points after electrochemotherapy (ECT) combined with gene electrotransfer of plasmid DNA encoding interleukin IL-12 (GET pIL-12) as noted at the top of the figure. Note the difference between tumors with complete response (CR) and tumors with progressive disease (PD) after therapy. Non-CR tumors (red circles) are filled with microbubbles, whereas CR tumors (blue circles) show no contrast enhancement immediately and 8 h after therapy.

The PE values were significantly lower in tumors with CR than in tumors with non-CR at all time points after therapy, except on day 3 (Figure 3). The difference in PE between CR and non-CR tumors was highest 8 h after therapy and gradually decreased in the following days but remained statistically significant on days 3 and 7. Note that the CR tumors showed no contrast enhancement immediately and 8 h after therapy; this finding is in contrast to non-CR tumors, which were still filled with microbubbles (Figure 4).

**Figure 5:**
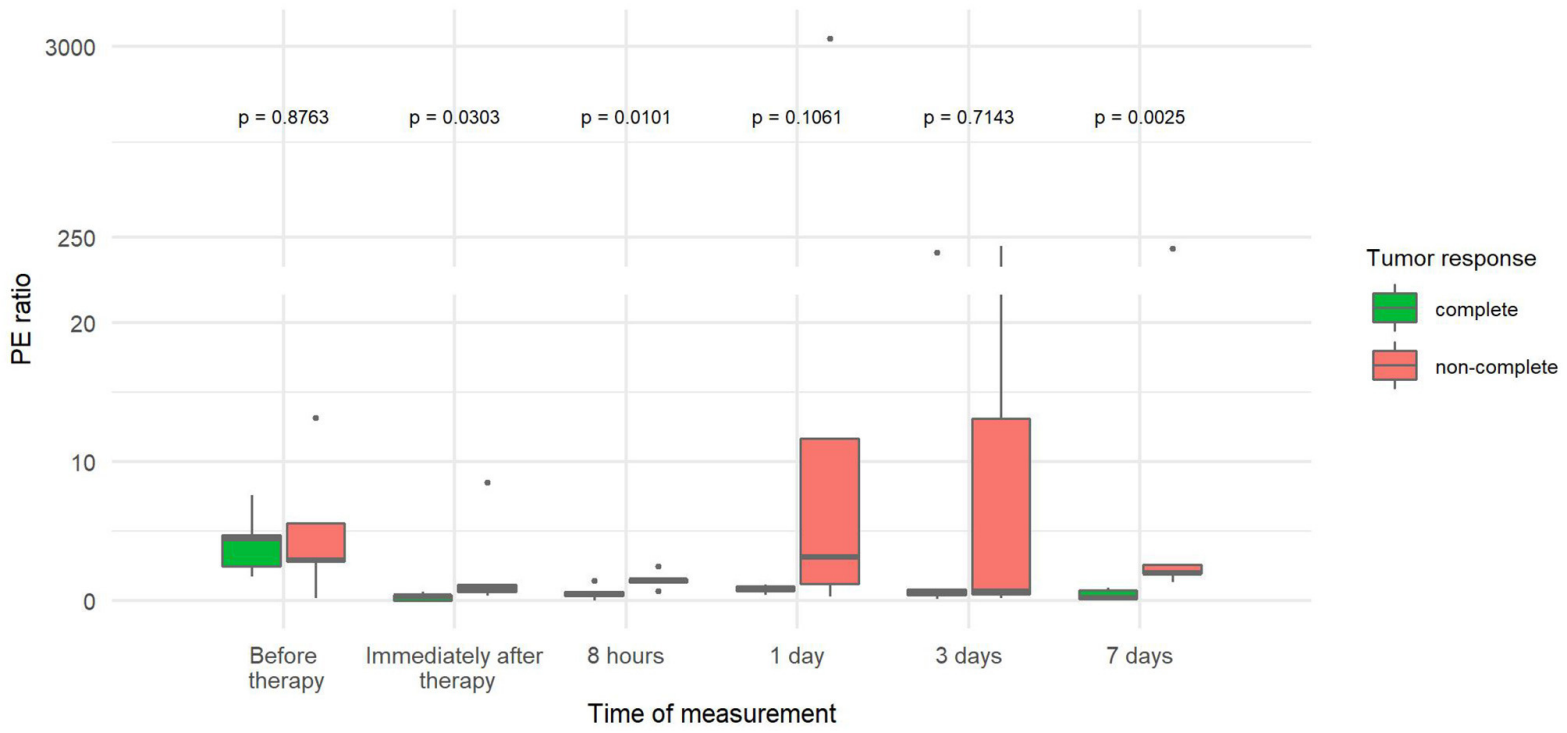
Comparison of ratios between tumor peak enhancement and reference peak enhancement (PE ratio) at different time points between canine tumors with a complete and non-complete response to electrochemotherapy (ECT) with gene electrotransfer of plasmid DNA encoding interleukin IL-12 (GET pIL-12). Note the *p*-values presented at the top of the image. PE ratio is significantly lower for complete response tumors compared with non-complete response tumors immediately after therapy and at 8 h and 7 days.

The PE ratio (Table 3) was significantly reduced in CR tumors compared to non-CR tumors immediately, 8 h and 7 days after therapy. The difference steadily increased over time, reaching a 45-fold decrease by day 7 (Figure 5).

**Figure 6:**
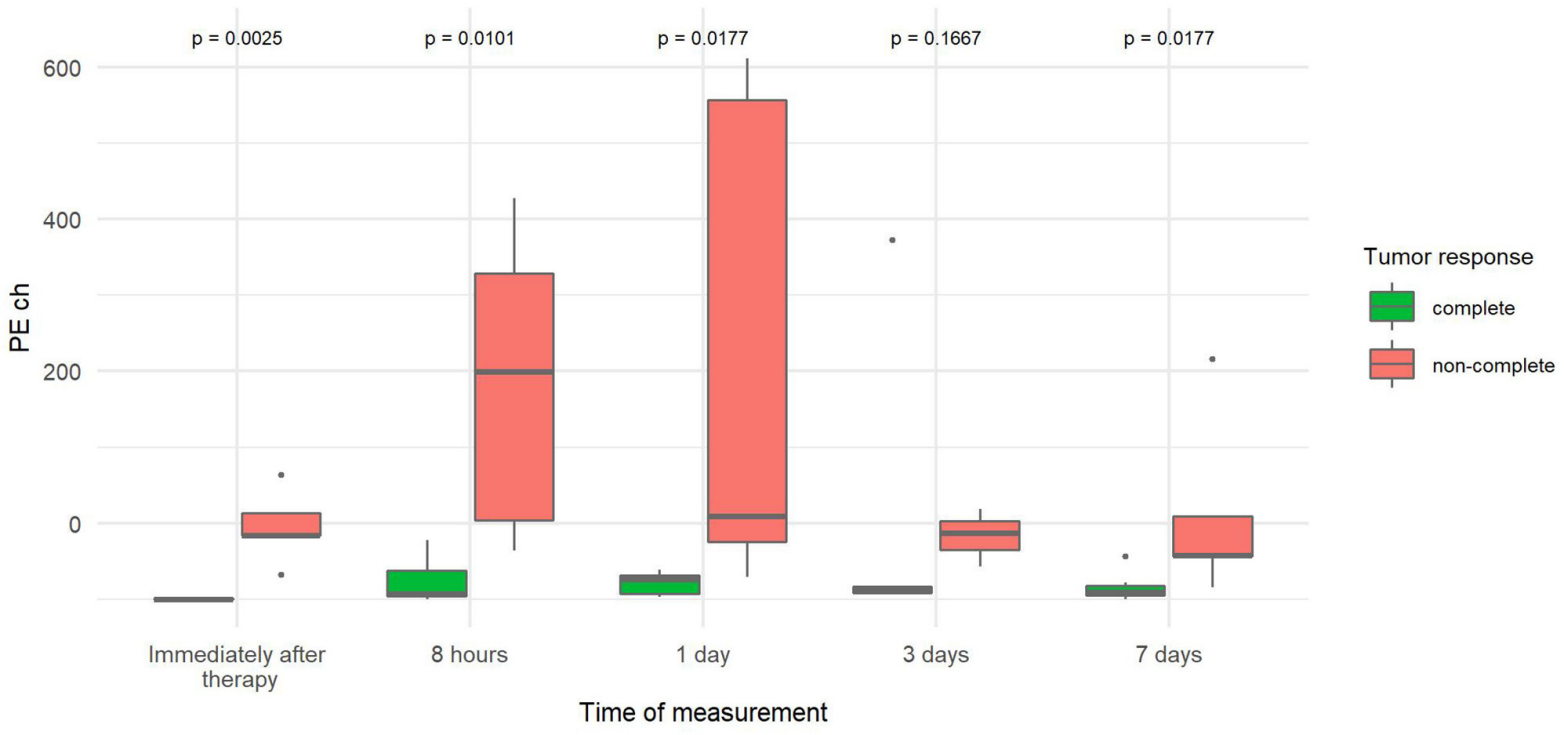
Comparison of the percentage change in peak enhancement of tumor (PE ch) at different time points between canine tumors with a complete and non-complete response to electrochemotherapy (ECT) with gene electrotransfer of plasmid DNA encoding interleukin IL-12 (GET pIL-12). Note the *p*-values presented at the top of the image. PE ch is significantly lower for complete response tumors compared with non-complete response tumors immediately after therapy and at 8 h and 7 days.

The PE change (Table 3) was significantly reduced in CR tumors at four time points: immediately, 8 h, and 1 and 7 days after therapy. The highest difference between the two tumor groups (20-fold decrease) was observed immediately after therapy. With time, the difference decreased but remained statistically significant (Figure 6).

**Figure 7:**
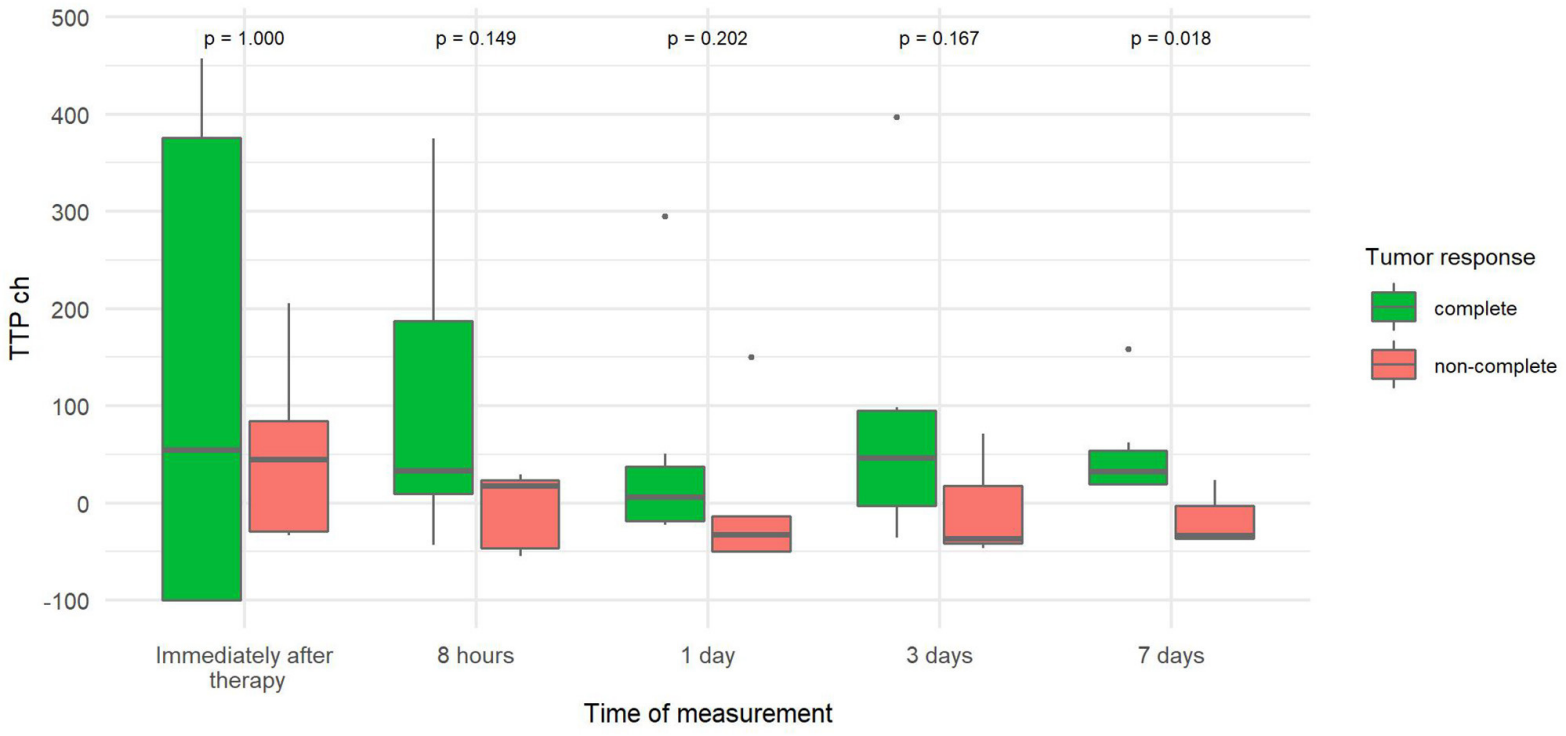
Comparison of the percentage change in time to peak of tumor (TTP ch) at different time points canine tumors with complete and non-complete response to electrochemotherapy (ECT) with gene electrotransfer of plasmid DNA encoding interleukin IL-12 (GET pIL-12). Note the *p*-values presented at the top of the image. TTP ch is significantly lower for complete response tumors compared with non-complete response tumors 7 days after therapy.

The percentage change in TTP from baseline TTP (TTP ch) was more than three times greater 7 days after therapy in tumors with CR than in tumors without CR (Figure 7) because TTP increased from baseline in CR tumors and decreased from baseline in non-CR tumors.

**Figure 8:**
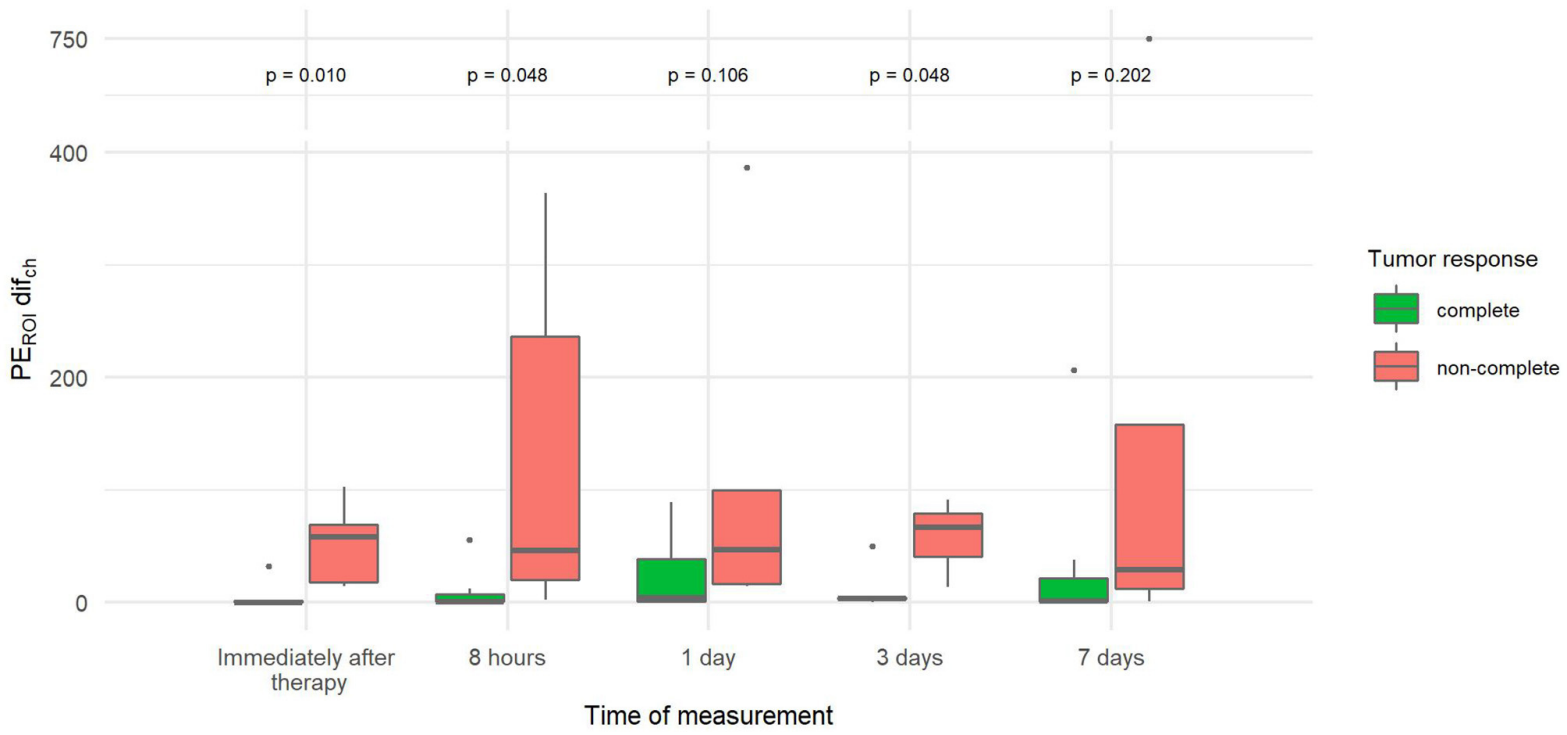
Comparison of the percentage difference in change of peak enhancement of the two tumor parts (PE_ROI_ dif_ch_) at different time points between canine tumors with a complete and non-complete response to electrochemotherapy (ECT) with gene electrotransfer of plasmid DNA encoding interleukin IL-12 (GET pIL-12). Note the *p*-values presented at the top of the image. PE_ROI_ dif_ch_ is significantly lower for complete response tumors compared with non-complete response tumors immediately after therapy and at 8 h and 3 days after therapy.

The percentage difference in the change in PE between the two parts of the tumor (PE_ROI_ dif_ch_), which describes tumor heterogeneity in PE, was more than 10–13 times lower immediately, 8 h and 3 days after therapy in tumors with CR compared to tumors with non-CR (Figure 8).

The parameters TTP, TTP_ROI_ dif, PE_ROI_ dif, and TTP ratio (Table 3) were not significantly different between CR and non-CR tumors.

When results for both groups (CR vs. non-CR tumors) regardless of the time were compared, PE, PE ratio, PE ch, TTP_ROI_ dif, and TTP_ROI_ dif_ch_ were significantly lower, and TTP ch was significantly higher in tumors with CR than in tumors with non-CR (Tables 3, 4).

**Table 4:** Comparison of overall results of dynamic contrast-enhanced ultrasound parameters (DCE-US) between tumors with complete response (CR) and tumors without complete response (non-CR) treated with electrochemotherapy (ECT) combined with gene electrotransfer (GET pIL-12).

**Parameter**		**Tumor response**		**Wilcoxon rank sum test**
		**CR**	**Non-CR**	
		**Median (1^**st**^ and 3^**rd**^ quartile)**	**Median (1^**st**^ and 3^**rd**^ quartile)**	***p*-value**
V	Volume of tumor before the treatment (cm^3^)	0.27 (0.06, 0.65)	0.37 (0.04, 1.10)	0.8072
PE	Peak enhancement	32.50 (14.87, 150.90)	312.56 (155.21, 803.50)	<0.0001
TTP	Time to peak	9.41 (6.70, 17.07)	8.90 (6.36, 13.42)	0.1242
*PE ratio*	Ratio between PE of the tumor and PE of the reference	0.64 (0.34, 1.10)	1.77 (0.84, 3.77)	<0.0001
*TTP ratio*	Ratio between TTP of the tumor and TTP of the reference	0.96 (0.57, 1.24)	0.79 (0.60, 1.29)	0.6926
*PE ch*	Percentage change in PE	−92.36 (−97.21, −79.13)	3.69 (−39.22, 131.43)	<0.0001
*TTP ch*	Percentage change in TTP	27.91 (−6.49, 94.75)	29.06 (−37.56, −26.51)	<0.0001
*PE*_*ROI*_ *dif*	Percentage difference in PE between ROI1 and ROI2	17.09 (5.81, 33.75)	30.20 (9.35, 38.07)	0.0892
*TTP*_*ROI*_ *dif*	Percentage difference in TTP between ROI1 and ROI2	5.71 (0.92, 15.69)	12.34 (7.02, 20.08)	0.0673
*PE*_*ROI*_ *dif*_*ch*_	Percentage difference in change of PE between ROI1 and ROI2	1.59 (0.29, 10.15)	47.01 (15.34, 100.99)	<0.0001
*TTP*_*ROI*_ *dif*_*ch*_	Percentage difference in change of TTP between ROI1 and ROI2	16.17 (11.06, 59.41)	50.00 (21.41, 130.35)	0.0577

## Discussion

This study shows a significant difference in DCE-US results between canine tumors that achieved a CR to ECT combined with GET pIL-12 and non-CR tumors. After therapy, perfusion of tumors was lower in the CR group, and perfusion heterogeneity was greater in non-CR tumors.

To the best of our knowledge, this is the first study to compare DCE-US results with treatment outcomes in canine tumors treated with ECT combined with GET pIL-12. Our results are consistent with human studies that have shown DCE-US results to be a useful tool for predicting the efficacy of other antiangiogenic treatments in metastatic renal cell carcinoma, advanced hepatocellular carcinoma, colorectal carcinoma, metastatic breast cancer, gastrointestinal stromal tumors, and metastatic melanoma (9–11, 14–16). Tumors included in this study were compared between the two groups regarding tumor perfusion and perfusion heterogeneity. Before therapy, no differences in perfusion parameters and perfusion heterogeneity were noted between CR and non-CR tumors. Perfusion decreased after therapy in all CR tumors, which is consistent with previously described vascular lock and vascular disrupting action of EP-based therapies (63–74). The decrease in tumor perfusion was greater in CR compared with non-CR tumors, supporting the assumption that therapy is more likely to be effective if it is reflected in immediate “vascular lock.” Furthermore, the difference remained statistically significant until day 7 with the exception of day 3. This finding is consistent with the expectation that the outcome of therapy is less likely to be favorable when therapy does not show antiangiogenic effects that result in decreased perfusion in the days after treatment. A similar trend was observed on day 3. However, due to owner non-compliance and thus missing data from one dog with three tumors, the difference was not statistically significant.

In a study of nine spontaneous canine tumors treated with GET pIL-12 (25), a significant decrease in the DCE-US parameters wash-in area under the curve describing relative blood volume and wash-in rate describing blood flow velocity was observed at days 8 and 35 compared to baseline, consistent with the results of our study. Similar parameters reflecting relative blood volume and blood flow velocity, namely PE and TTP, respectively, were investigated by the same authors in a study of five dogs treated with GET pIL-12 in combination with metronomic cyclophosphamide (30). They observed a significant decrease in PE and prolonged TTP 35 days after treatment, which is consistent with our results, while in contrast to our results, an increase in PE was observed 8 days after treatment. The difference in the dynamics of PE can most likely be attributed to the fact that the study by Cicchelero et al. (30) investigated GET pIL-12 (without ECT), which does not induce a “vascular disrupting effect,” i.e., cytotoxic effect of chemotherapeutic drugs on vascular endothelial cells (67, 68, 72) that can be observed after treatment with ECT. Treatment with GET pIL-12 results in a much less pronounced antitumor effect (27), also observed in this study, as no clinically relevant outcome was observed (30). However, it is likely that the decrease in PE and prolongation of TTP 35 days after treatment is a consequence of the antiangiogenic effect of gene therapy. An additional difference between our studies is also the fact that the latter study (30) included five different tumor types and DCE-US was performed only at three time points, i.e., before therapy and at days 8 and 35; our data cannot be directly compared because they were collected at different time points and after different treatments.

A common feature of malignant tumors compared with non-malignant tumors is rapid wash-in (78, 79), which may be associated with shorter TTP. In our study, 7 days after therapy, the percentage change in TTP was greater in CR tumors, i.e., the time to peak increased significantly compared with the measurement before therapy, which may indicate that the tumors became “less malignant” with the treatment performed. These results are similar to those of human studies examining various chemotherapeutic antiangiogenic treatments, in which TTP and wash-in rate decreased after therapy (10, 17, 80). In a study of the efficacy of sorafenib treatment of metastatic renal cancer in humans, the correlation between treatment outcome and the percentage of perfused tissue decreased 3 weeks after treatment (15). In contrast, in studies evaluating DCE-US after radiotherapy for spontaneous tumors in dogs (18, 81), DCE-US results were not predictive of disease outcome. This difference can be explained by an important difference in the mechanism of action of radiotherapy compared to ECT combined with GET pIL-12. Radiotherapy is less efficient in regions of lower perfusion due to the resistance of hypoxic cells to treatment, whereas the presented EP-based treatment exerts antiangiogenic and cytotoxic effects that do not depend on cell oxygenation.

Perfusion heterogeneity is a hallmark of malignant tumors and provides valuable information for discriminating between malignant and benign lesions (82). We demonstrated a significant difference in perfusion heterogeneity between the two different groups based on the clinical response: tumors reaching CR were less heterogeneously perfused than non-CR tumors. This is an important finding as it indicates a possible predictive value of perfusion heterogeneity in therapies based on antiangiogenic effects. Similar results were obtained by DCE-CT in human hepatic neoplasia treated with antiangiogenic therapy; reduced perfusion heterogeneity correlated with better local tumor control and longer survival (83). In contrast, in human cervical cancer treated with radiotherapy and chemotherapy and evaluated with dynamic contrast-enhanced magnetic resonance imaging (DCE-MRI), decreased perfusion heterogeneity correlated with poorer outcomes due to a lower response to radiotherapy in those parts of the tumor that are hypoxic due to decreased perfusion (82). Therefore, perfusion and perfusion heterogeneity evaluated with DCE-US appear to be useful for predicting the results of antiangiogenic treatments but cannot be used for all types of anticancer therapy.

Investigation of perfusion is an appealing method to predict outcome in tumors treated with antiangiogenic therapies. Our results show that DCE-US is safe for the patient; no adverse effects of contrast administration were noted despite repeated administration. Furthermore, it is a simple method to assess tumor perfusion that can be easily repeated during and after treatment and, based on our study, is associated with treatment outcome in canine tumors treated with a combination of ECT and GET pIL-12. The next clinically applicable step would be to investigate the predictive ability of DCE-US to distinguish between tumors that were successfully treated and those in which the therapy is unlikely to lead to a complete response after EP-based therapies. This information would allow a clinician to perform such a therapeutic procedure on a patient to evaluate if and when the therapy should be repeated, preferably in the early stages of the treatment when targeted therapy adjustments are generally more effective. This was not possible in our study given that a much larger number of patients are needed for logistic regression models. If a predictive value is to be confirmed, appropriate cutoff values for DCE-US parameters should be ascertained to make effective decisions about prognosis and the need for repeated or additional therapy in individual patients.

## Data Availability Statement

The original contributions presented in the study are included in the article/supplementary material, further inquiries can be directed to the corresponding author/s.

## Ethics Statement

The animal study was reviewed and approved by National Ethics Committee at the Administration of the Republic of Slovenia for Food Safety, Veterinary, and Plant Protection (U34401-24/2014/4). Written informed consent was obtained from the owners for the participation of their animals in this study.

## Author Contributions

DP, MC, GS, NT, and MB: conception and design. MB, NB, NM, and DP: acquisition of data. MB, NB, NM, TK, DP, and SKB: analysis and interpretation of data. MB, NB, DP, and SKB: drafting the article. GS, MC, NT, DP, and SKB: revising the article for intellectual content. All authors: final approval of the completed article.

## Conflict of Interest

The authors declare that the research was conducted in the absence of any commercial or financial relationships that could be construed as a potential conflict of interest.

## References

[1] Donelly EF Geng L Wojcicki WE Fleischer AC Hallahan DE. Quantified power Doppler US of tumor blood flow correlates with microscopic quantification of tumor blood vessels. Radiology. (2001) 219:166–70. 10.1148/radiology.219.1.r01ap38166 11274552

[2] Zhou JH Cao LH Liu JB Zheng W Liu M Luo RZ. Quantitative assessment of tumor blood flow in mice after treatment with different doses of an antiangiogenic agent with contrast-enhanced destruction-replenishment US. Radiology. (2011) 259:406–13. 10.1148/radiol.10101339 21292869

[3] Zhou JH Cao LH Zheng W Liu M Han F Li AH. Contrast-enhanced gray-scale ultrasound for quantitative evaluation of tumor response to chemotherapy: preliminary results with a mouse hepatoma model. AJR Am J Roentgenol. (2011) 196:W13–7. 10.2214/AJR.10.4734 21178025

[4] Sedelaar JP van Leenders GJ Hulsbergen-van de Kaa CA van der Poel HG van der Laak JA Debruyne FM. Microvessel density: correlation between contrast ultrasonography and histology of prostate cancer. Eur Urol. (2001) 40:285–93. 10.1159/000049788 11684844

[5] Leng X Huang G Ma F Yao L. Regional Contrast-Enhanced Ultrasonography (DCE-US) characteristics of breast cancer and correlation with Microvessel Density (MVD). Med Sci Monit. (2017) 23:3428–36. 10.12659/MSM.901734 28708818 PMC5523962

[6] Li X Li Y Zhu Y Fu L Liu P. Association between enhancement patterns and parameters of contrast-enhanced ultrasound and microvessel distribution in breast cancer. Oncol Lett. (2018) 15:5643–9. 10.3892/ol.2018.8078 29556301 PMC5844090

[7] Ohlerth S Wergin M Bley CR Del Chicca F Laluhová D Hauser B. Correlation of quantified contrast-enhanced power Doppler ultrasonography with immunofluorescent analysis of microvessel density in spontaneous canine tumours. Vet J. (2010) 183:58–62. 10.1016/j.tvjl.2008.08.026 18922713

[8] Lassau N Chebil M Chami L. A new functional imaging technique for the early functional evaluation of antiangiogenic treatment: dynamic contrast-enhanced ultrasonography (DCE-US). Target Oncol. (2008) 3:111–7. 10.1007/s11523-008-0081-x 20379790

[9] Lassau N Chebil M Chami L Bidault S Girard E Roche A. Dynamic contrast-enhanced ultrasonography (DCE-US): a new tool for the early evaluation of antiangiogenic treatment. Target Oncol. (2010) 5:53–8. 10.1007/s11523-010-0136-7 20379790

[10] Lassau N Koscielny S Chami L Chebil M Benatsou B Roche A. Advanced hepatocellular carcinoma: early evaluation of response to bevacizumab therapy at dynamic contrast-enhanced US with quantification - preliminary results. Radiology. (2011) 258:291–300. 10.1148/radiol.10091870 20980447

[11] Lassau N Chapotot L Benatsou B Vilgrain V Kind M Lacroix J. Standardization of dynamic contrast-enhanced ultrasound for the evaluation of antiangiogenic therapies: the French multicenter Support for Innovative and Expensive Techniques Study. Invest Radiol. (2012) 47:711–66. 10.1097/RLI.0b013e31826dc255 23095862

[12] Lassau N Cosgrove D Armand JP. Early evaluation of targeted drugs using dynamic contrast-enhanced ultrasonography for personalized medicine. Future Oncol. (2012) 8:1215–8. 10.2217/fon.12.114 23130922

[13] Lassau N Bonastre J Kind M Vilgrain V Lacroix J Cuinet M. Validation of dynamic contrast-enhanced ultrasound in predicting outcomes of antiangiogenic therapy for solid tumors: the French multicenter support for innovative and expensive techniques study. Invest Radiol. (2014) 49:794–800. 10.1097/RLI.0000000000000085 24991866 PMC4222794

[14] Lassau N Coiffier B Kind M Vilgrain V Lacroix J Cuinet M. Selection of an early biomarker for vascular normalization using dynamic contrast-enhanced ultrasonography to predict outcomes of metastatic patients treated with bevacizumab. Ann Oncol. (2016) 27:1922–8. 10.1093/annonc/mdw280 27502701 PMC5035788

[15] Lamuraglia M Escudier B Chami L Schwartz B Leclere J Roche A. To predict progression-free survival and overall survival in metastatic renal cancer treated with sorafenib: pilot study using dynamic contrast-enhanced Doppler ultrasound. Eur J Cancer. (2006) 42:2472–9. 10.1016/j.ejca.2006.04.023 16965911

[16] Escudier B Lassau N Angevin E Soria JC Chami L Lamuraglia M. Phase I trial of sorafenib in combination with IFN alpha-2a in patients with unresectable and/or metastatic renal cell carcinoma or malignant melanoma. Clin Cancer Res. (2007) 13:1801–9. 10.1158/1078-0432.CCR-06-1432 17363536

[17] Kim Y Kim SH Song BJ Kang BJ Yim KI Lee A. Early prediction of response to neoadjuvant chemotherapy using Dynamic Contrast-Enhanced MRI and Ultrasound in breast cancer. Korean J Radiol. (2018) 19:682–91. 10.3348/kjr.2018.19.4.682 29962874 PMC6005946

[18] Rohrer Bley C Laluhova D Roos M Kaser-Hotz B Ohlerth S. Correlation of pretreatment polarographically measured oxygen pressures with quantified contrast-enhanced power doppler ultrasonography in spontaneous canine tumors and their impact on outcome after radiation therapy. Strahlenther Onkol. (2009) 185:756–62. 10.1007/s00066-009-1988-6 19899010

[19] Nierman KJ Fleischer AC Huamani J Yankeelov TE Kim DW Wilson WD. Measuring tumor perfusion in control and treated murine tumors: correlation of microbubble contrast-enhanced sonography to dynamic contrast-enhanced magnetic resonance imaging and fluorodeoxyglucose positron emission tomography. J Ultrasound Med. (2007) 26:749–56. 10.7863/jum.2007.26.6.749 17526606

[20] Denham SL Alexander LF Robbin ML. Contrast-enhanced ultrasound: practical review for the assessment of hepatic and renal lesions. Ultrasound Q. (2016) 32:116–25. 10.1097/RUQ.0000000000000182 27233070

[21] Fetzer DT Rafailidis V Peterson C Grant EG Sidhu P Barr RG. Artifacts in contrast-enhanced ultrasound: a pictorial essay. Abdom Radiol. (2018) 43:977–97. 10.1007/s00261-017-1417-8 29198008

[22] Erlichman DB Weiss A Koenigsberg M Stein MW. Contrast enhanced ultrasound: a review of radiology applications. Clin Imaging. (2019) 60:209–15. 10.1016/j.clinimag.2019.12.013 31927496

[23] Seiler GS Brown JC Reetz JA Taeymans O Bucknoff M Rossi F. Safety of contrast-enhanced ultrasonography in dogs and cats: 488 cases (2002-2011). J Am Vet Med Assoc. (2013) 242:1255–9. 10.2460/javma.242.9.1255 23600783

[24] Dolan MS Gala SS Dodla S Abdelmoneim SS Xie F Cloutier D. Safety and efficacy of commercially available ultrasound contrast agents for rest and stress echocardiography: a multicenter experience. J Am Coll Cardiol. (2009) 53:32–8. 10.1016/j.jacc.2008.08.066 19118722

[25] Cicchelero L Denies S Vanderperren K Stock E Van Brantegem L de Rooster H. Intratumoural interleukin 12 gene therapy stimulates the immune system and decreases angiogenesis in dogs with spontaneous cancer. Vet Comp Oncol. (2017) 15:1187–205. 10.1111/vco.12255 27506827

[26] Salvadori C Svara T Rocchigiani G Millanta F Pavlin D Cemazar M. Effects of electrochemotherapy with cisplatin and peritumoral il-12 gene electrotransfer on canine mast cell tumors: a histopathologic and immunohistochemical study. Radiol Oncol. (2017) 51:286–94. 10.1515/raon-2017-0035 28959165 PMC5611993

[27] Pavlin D Cemazar M Cör A Sersa G Pogačnik A Tozon N. Electrogene therapy with interleukin-12 in canine mast cell tumors. Radiol Oncol. (2011) 45:30–9. 10.2478/v10019-010-0041-9 22933932 PMC3423723

[28] Milevoj N Lampreht Tratar U Nemec A Brozic A Znidar K Sersa G. A combination of electrochemotherapy, gene electrotransfer of plasmid encoding canine IL-12 and cytoreductive surgery in the treatment of canine oral malignant melanoma. Res Vet Sci. (2019) 122:40–9. 10.1016/j.rvsc.2018.11.001 30453179

[29] Milevoj N Tozon N Licen S Lampreht Tratar U Sersa G Cemazar M. Health-related quality of life in dogs treated with electrochemotherapy and/or interleukin-12 gene electrotransfer. Vet Med Sci. (2020) 6:290–8. 10.1002/vms3.232 31910331 PMC7397887

[30] Cicchelero L Denies S Vanderperren K Stock E Van Brantegem L de Rooster H. Immunological, anti-angiogenic and clinical effects of intratumoral interleukin 12 electrogene therapy combined with metronomic cyclophosphamide in dogs with spontaneous cancer: a pilot study. Cancer Lett. (2017) 400:205–18. 10.1016/j.canlet.2016.09.015 27693635

[31] Cutrera J King G Jones P Kicenuik K Gumpel E Xia X. Safe and effective treatment of spontaneous neoplasms with interleukin 12 electro-chemo-gene therapy. J Cell Mol Med. (2015) 19:664–75. 10.1111/jcmm.12382 25628149 PMC4369822

[32] Cemazar M Jarm T Sersa G. Cancer electrogene therapy with interleukin-12. Curr Gene Ther. (2010) 10:300–11. 10.2174/156652310791823425 20560875

[33] Cemazar M Ambrozic Avgustin J Pavlin D Sersa G Poli A Levacic Krhač A. Efficacy and safety of electrochemotherapy combined with peritumoral IL-12 gene electrotransfer of canine mast cell tumours. Vet Comp Oncol. (2017) 15:641–54. 10.1111/vco.12208 26840222

[34] Cemazar M Tamzali Y Sersa G Tozon N Mir LM Miklavcic D. Electrochemotherapy in veterinary oncology. J Vet Intern Med. (2008) 22:826–31. 10.1111/j.1939-1676.2008.0117.x 18537879

[35] Nemec A Milevoj N Lampreht Tratar U Sersa G Cemazar M Tozon N. Electroporation-based treatments in small animal veterinary oral and maxillofacial oncology. Front Vet Sci. (2020) 7:575911. 10.3389/fvets.2020.575911 33134356 PMC7550461

[36] Reed SD Fulmer A Buckholz J Zhang B Cutrera J Shiomitsu K. Bleomycin/interleukin-12 electrochemogenetherapy for treating naturally occurring spontaneous neoplasms in dogs. Cancer Gene Ther. (2010) 17:571–8. 10.1038/cgt.2010.13 20414325

[37] Impellizeri J Aurisicchio L Forde P Soden DM. Electroporation in veterinary oncology. Vet J. (2016) 217:18–25. 10.1016/j.tvjl.2016.05.015 27810205

[38] Kodre V Cemazar M Pecar J Sersa G Cor A Tozon N. Electrochemotherapy compared to surgery for treatment of canine mast cell tumours. In Vivo. (2009) 23:55–62. 19368125

[39] Spugnini EP Di Tosto G Salemme S Pecchia L Fanciulli M Baldi A. Electrochemotherapy for the treatment of recurring aponeurotic fibromatosis in a dog. Can Vet J. (2013) 54:606–9. 24155455 PMC3659460

[40] Spugnini EP Vincenzi B Citro G Dotsinsky I Mudrov T Baldi A 2011. Evaluation of cisplatin as an electrochemotherapy agent for the treatment of incompletely excised mast cell tumors in dogs. J Vet Intern Med. (2011) 25:407–11. 10.1111/j.1939-1676.2011.0678.x 21382075

[41] Tellado MN Maglietti FH Michinski SD Marshall GR Signori E. Predictive factors of response to electrochemotherapy in canine oral malignant melanoma. Radiol Oncol. (2020) 54:68–78. 10.2478/raon-2020-0014 32187017 PMC7087426

[42] Tozon N Pavlin D Sersa G Dolinsek T Cemazar M. Electrochemotherapy with intravenous bleomycin injection: an observational study in superficial squamous cell carcinoma in cats. J Feline Med Surg. (2014) 16:291–9. 10.1177/1098612X13507071 24127456 PMC11383118

[43] Racnik J Svara T Zadravec M Gombac M Cemazar M Sersa G. Electrochemotherapy with bleomycin of different types of cutaneous tumours in a ferret (Mustela Putorius Furo). Radiol Oncol. (2017) 52:98–104. 10.1515/raon-2017-0057 29520211 PMC5839087

[44] Tamzali Y Borde L Rols MP Golzio M Lyazrhi F Teissie J. Successful treatment of equine sarcoids with cisplatin electrochemotherapy: a retrospective study of 48 cases. Equine Vet J. (2012) 44:214–20. 10.1111/j.2042-3306.2011.00425.x 21793876

[45] Tozon N Kramaric P Kos Kadunc V Sersa G Cemazar M. Electrochemotherapy as a single or adjuvant treatment to surgery of cutaneous sarcoid tumours in horses: a 31-case retrospective study. Vet Rec. (2016) 179:1–6. 10.1136/vr.103867 27758950

[46] Glass LF Pepine ML Fenske NA Jaroszeski M Reintgen DS Heller R. Bleomycin-mediated electrochemotherapy of metastatic melanoma. Arch Dermatol. (1996) 132:1353–7. 10.1001/archderm.1996.03890350095015 8915314

[47] Pavlin D Cemazar D Sersa G Tozon N. IL-12 based gene therapy in veterinary medicine. J Transl Med. (2012) 10:234–44. 10.1186/1479-5876-10-234 23171444 PMC3543347

[48] Sedlar A Dolinšek T Markelc B Prosen L Kranjc S Bosnjak M. Potentiation of electrochemotherapy by intramuscular IL-12 gene electrotransfer in murine sarcoma and carcinoma with different immunogenicity. Radiol Oncol. (2012) 46:302–11. 10.2478/v10019-012-0044-9 23412658 PMC3572893

[49] Kishida T Asada H Itokawa Y Yasutomi K Shin-Ya M Gojo J. Electrochemo-gene therapy of cancer: intratumoral delivery of interleukin-12 gene and bleomycin synergistically induced therapeutic immunity and suppressed subcutaneous and metastatic melanomas in mice. Mol Ther. (2003) 8:738–45. 10.1016/j.ymthe.2003.08.002 14599806

[50] Maglietti F Tellado M De Robertis M Michinski S Fernandez J Signori E. Electroporation as the immunotherapy strategy for cancer in veterinary medicine: state of the art in Latin America. Vaccines (Basel). (2020) 8:537. 10.3390/vaccines8030537 32957424 PMC7564659

[51] Heller R Jaroszeski MJ Reintgen DS Puleo CA DeConti RC Gilbert RA. Treatment of cutaneous and subcutaneous tumors with electrochemotherapy using intralesional bleomycin. Cancer. (1998) 83:148–157. 10.1002/(SICI)1097-0142(19980701)83:1<148::AID-CNCR20>3.0.CO;2-W 9655305

[52] Sersa G Stabuc B Cemazar M Miklavcic D Rudolf Z. Electrochemotherapy with cisplatin: clinical experience in malignant melanoma patients. Clin Cancer Res. (2000) 6:863–7. 10741708

[53] Byrne CM Thompson JF Johnston H Hersey P Quinn MJ Michael Hughes T. Treatment of metastatic melanoma using electroporation therapy with bleomycin (electrochemotherapy). Melanoma Res. (2005) 15:45–51. 10.1097/00008390-200502000-00008 15714120

[54] Gaudy C Richard MA Folchetti G Bonerandi JJ Grob JJ. Randomized controlled study of electrochemotherapy in the local treatment of skin metastases of melanoma. J Cutan Med Surg. (2006) 10:115–21. 10.2310/7750.2006.00037 17241586

[55] Marty M Serša G Rémi Garbay J Gehl J Collins CG Snoj M. Electrochemotherapy - an easy, highly effective and safe treatment of cutaneous and subcutaneous metastases: results of ESOPE (European Standard Operating Procedures of Electrochemotherapy) study. EJC Suppl. (2006) 4:3–13. 10.1016/j.ejcsup.2006.08.002

[56] Mir LM Gehl J Sersa G Collins CG Garbay JR Billard V. Standard operating procedures of the electrochemotherapy: instructions for the use of bleomycin or cisplatin administered either systemically or locally and electrical pulses delivered by Cliniporator by means of invasive or non-invasive electrodes. Eur J Cancer Suppl. (2006) 4:14–25. 10.1016/j.ejcsup.2006.08.003

[57] Bertino G Serša G De Terlizzi F Occhini A Plaschke CC Groselj A. European research on electrochemotherapy in head and neck cancer (EURECA) project: results of the treatment of skin cancer. Eur J Cancer. (2016) 63:41–52. 10.1016/j.ejca.2016.05.001 27267144

[58] Campana LG Marconato R Valpione S Galuppo S Alaibac M Rossi CR. Basal cell carcinoma: 10-year experience with electrochemotherapy. J Transl Med. (2017) 15:122. 10.1186/s12967-017-1225-5 28569161 PMC5452531

[59] Colletti L Battaglia V De Simone P Turturici L Bartolozzi C Filipponi F. Safety and feasibility of electrochemotherapy in patients with unresectable colorectal liver metastases: a pilot study. Int J Surg. (2017) 44:26–32. 10.1016/j.ijsu.2017.06.033 28624558

[60] Djokic M Cemazar M Popovic P Kos B Dezman R Bosnjak M. Electrochemotherapy as treatment option for hepatocellular carcinoma, a prospective pilot study. Eur J Surg Oncol. (2018) 44:651–7. 10.1016/j.ejso.2018.01.090 29402556

[61] Edhemovic I Brecelj E Gasljevic G Marolt Music M Gorjup V Mali B. Intraoperative electrochemotherapy of colorectal liver metastases. J Surg Oncol. (2014) 110:320–7. 10.1002/jso.23625 24782355

[62] Ruers T van Coevorden F Punt CJA Pierie JPEN Borel-Rinkes I Ledermann JA. Local treatment of unresectable colorectal liver metastases: results of a randomized phase II trial. J Natl Cancer Inst. (2017) 109:djx015. 10.1093/jnci/djx015 28376151 PMC5408999

[63] Edhemovic I Brecelj E Cemazar M Boc N Trotovsek B Djokic M. Intraoperative electrochemotherapy of colorectal liver metastases: a prospective phase II study. Eur J Surg Oncol. (2020) 46:1628–33. 10.1016/j.ejso.2020.04.037 32387070

[64] Tafuto S von Arx C De Divitiis Maura CT Palaia R Albino V. Electrochemotherapy as a new approach on pancreatic cancer and on liver metastases. Int J Surg. (2015) 21:S78–S82. 10.1016/j.ijsu.2015.04.095 26123385

[65] Granata V Fusco R Piccirillo M Palaia R Petrillo A Lastoria S. Electrochemotherapy in locally advanced pancreatic cancer: Preliminary results. Int J Surg. (2015) 18:230–6. 10.1016/j.ijsu.2015.04.055 25917204

[66] Bimonte S Leongito M Granata V Barbieri A Vecchio VD Falco M. Electrochemotherapy in pancreatic adenocarcinoma treatment: pre-clinical and clinical studies. Radiol Oncol. (2016) 50:14–20. 10.1515/raon-2016-0003 27069445 PMC4825336

[67] Sersa G Cemazar M Miklavcic D Chaplin DJ. Tumor blood flow modifying effect of electrochemotherapy with bleomycin. Anticancer Res. (1999) 19:4017–22. 10628347

[68] Sersa G Cemazar M Miklavcic D. Tumor blood flow modifying effects of electrochemotherapy: a potential vascular targeted mechanism. Radiol Oncol. (2003) 37:43–8.

[69] Sersa G Jarm T Kotnik T Coer A Podkrajsek M Sentjurc M. Vascular disrupting action of electroporation and electrochemotherapy with bleomycin in murine sarcoma. Br J Cancer. (2008) 98:388–98. 10.1038/sj.bjc.6604168 18182988 PMC2361464

[70] Jarm T Cemazar M Miklavcic D Sersa G. Antivascular effects of electrochemotherapy: implications in treatment of bleeding metastases. Expert Rev Anticancer Ther. (2010) 10:729–46. 10.1586/era.10.43 20470005

[71] Gehl J Skovsgaard T Mir LM. Vascular reactions to in vivo electroporation: characterization and consequences for drug and gene delivery. Biochim Biophys Acta. (2002) 1569:51–8. 10.1016/S0304-4165(01)00233-1 11853957

[72] Markelc B Sersa G Cemazar M. Differential mechanisms associated with vascular disrupting action of electrochemotherapy: intravital microscopy on the level of single normal and tumor blood vessels. PLoS One. (2013) 8:e59557. 10.1371/journal.pone.0059557 23555705 PMC3608732

[73] Boc N Edhemovic I Kos B Marolt Music M Brecelj E Bosnjak M. Ultrasonographic changes in the liver tumors as indicators of adequate tumor coverage with electric field for effective electrochemotherapy. Radiol Oncol. (2018) 52:383–391. 10.2478/raon-2018-0041 30352044 PMC6287182

[74] Brloznik M Boc N Sersa G Zmuc J Gasljevic G Seliskar A. Radiological findings of porcine liver after electrochemotherapy with bleomycin. Radiol Oncol. (2019) 53:415–26. 10.2478/raon-2019-0049 31600140 PMC6884938

[75] Schwarz LH Litiere S de Vries E Ford R Gwyther S Mandrekar S. RECIST 1.1 - update and clarification: from the RECIST Committee. Eur J Cancer. (2016) 62:132–7. 10.1016/j.ejca.2016.03.081 27189322 PMC5737828

[76] Seymour K Bogaerts J Perrone A Ford R Schwartz LH Mandrekar S. iRECIST: guidelines for response criteria for use in trials testing immunotherapeutics. Lancet Oncol. (2017) 18:e143–e52. 10.1016/S1470-2045(17)30074-8 28271869 PMC5648544

[77] R Core Team. R: A Language and Environment for Statistical Computing, version 3.5.0. Vienna: R Foundation for Statistical Computing (2019). Available online at: https://www.R-project.org (November 15, 2020).

[78] Kong WT Wang WP Huang BJ Ding H Mao F. Value of wash-in and wash-out time in the diagnosis between hepatocellular carcinoma and other hepatic nodules with similar vascular pattern on contrast-enhanced ultrasound. J Gastroenterol Hepatol. (2014) 29:576–80. 10.1111/jgh.12394 24118042

[79] Li YJ Wen G Wang Y Wang DX Yang L Deng YJ. Perfusion heterogeneity in breast tumors for assessment of angiogenesis. J Ultrasound Med. (2013) 32:1145–55. 10.7863/ultra.32.7.1145 23804337

[80] Lassau N Koscielny S Albiges L Chami L Benatsou B Chebil M. Metastatic renal cell carcinoma treated with sunitinib: early evaluation of treatment response using dynamic contrast-enhanced ultrasonography. Clin Cancer Res. (2010) 16:1216–25. 10.1158/1078-0432.CCR-09-2175 20145174

[81] Ohlerth S Bley CR Laluhová D Roos M Kaser-Hotz B. Assessment of changes in vascularity and blood volume in canine sarcomas and squamous cell carcinomas during fractionated radiation therapy using quantified contrast-enhanced power Doppler ultrasonography: a preliminary study. Vet J. (2010) 186:58–63. 10.1016/j.tvjl.2009.07.006 19692273

[82] Mayr NA Huang Z Wang JZ Lo SS Fan JM Grecula JC. Characterizing tumor heterogeneity with functional imaging and quantifying high-risk tumor volume for early prediction of treatment outcome: cervical cancer as a model. Int J Radiat Oncol Biol Phys. (2012) 83:972–9. 10.1016/j.ijrobp.2011.08.011 22208967 PMC4373343

[83] Hayano K Lee SH Yoshida H Zhu AX Sahani DV. Fractal analysis of CT perfusion images for evaluation of antiangiogenic treatment and survival in hepatocellular carcinoma. Acad Radiol. (2014) 21:654–60. 10.1016/j.acra.2014.01.020 24703479

